# Severe chest and back pain due to extra-pulmonary emphysema above 5,000 m: a series of case reports

**DOI:** 10.3389/fmed.2025.1640035

**Published:** 2025-10-06

**Authors:** Zhixin Gan, Jialin Wu, Jingdu Tian, Xiaoli Han, Qian Yang, Lei Zhang, Xi Yang, Xiaobo Han

**Affiliations:** General Hospital of Xinjiang Military Commands, Urumqi, China

**Keywords:** high altitude, extra-pulmonary emphysema, subcutaneous emphysema, mediastinal emphysema, management

## Abstract

**Background:**

Extra-pulmonary emphysema is characterized by the presence of gas within loose soft tissues outside the lungs. This condition is frequently mistaken for acute cardiovascular diseases, leading to potential misdiagnosis. Enhancing clinical recognition of extra-pulmonary emphysema is critical for improving patient outcomes at high altitude. Notably, hyperbaric oxygen therapy has shown promise as a treatment modality for this condition.

**Object:**

To evaluate the feasibility of early detection and hyperbaric oxygen therapy for extra-pulmonary emphysema in high-altitude regions.

**Method:**

A retrospective analysis was conducted on non-trauma patients presenting with chest and back pain in areas exceeding 5,000 meters in altitude from May to December 2024. Patients with a confirmed diagnosis of extra-pulmonary emphysema based on computed tomography (CT) imaging were included. Clinical data and treatment details were collected, and prognostic outcomes were monitored.

**Results:**

Nine young male patients, averaging 23.56 ± 5.27 years, presented with symptoms of neck and chest pain. None had a history of chronic disease, surgery, or trauma. Initial misdiagnosis occurred in seven cases, with only two correctly identified as simple extra-pulmonary emphysema. Eight patients showed significantly improvement following hyperbaric oxygen therapy at 2.0 atmospheres absolute (ATA).

**Conclusion:**

Recognizing extra-pulmonary emphysema at high altitude is crucial, especially severe mediastinal emphysema. Hyperbaric oxygen therapy may serve as an effective treatment option for this condition.

## Introduction

Extra-pulmonary emphysema is defined as gas accumulation within soft tissues, particularly in areas such as the neck and chest. This condition can be classified into subcutaneous emphysema (SE) and mediastinal emphysema (ME), depending on the location of the gas infiltration. The etiology of extra-pulmonary emphysema is multifactorial, encompassing trauma from blunt or penetrating injuries, surgical interventions, and infections. Furthermore, any condition that creates a pressure differential between the alveoli and the surrounding vascular interstitial tissue can precipitate this phenomenon (1–3).

The pathophysiology of extra-pulmonary emphysema is explained by the Macklin effect, in which elevated intrathoracic pressure leads to alveolar rupture, permitting gas to escape into the perivascular interstitium and subsequently dissect into the mediastinum (4). Common triggers include violent coughing, breath-holding, strenuous exercise, acute asthma attacks, and traumatic injury. This mechanism is of particular relevance in high-altitude settings. Hypoxia can induce pulmonary vasoconstriction and endothelial injury, thereby facilitating the entry of gas into perivascular and peribronchial sheaths. Furthermore, the compensatory deep and labored breathing characteristic of high-altitude exposure exacerbates the pressure gradient between alveoli and vascular structures, increasing the risk of alveolar rupture (5). Physical exertion under such conditions can further raise intrathoracic pressure, promoting the development of extra-pulmonary emphysema.

Diagnosis of extra-pulmonary emphysema typically involves clinical assessment of swelling and crepitus over affected areas, while imaging studies are essential for evaluating deep tissue involvement (6). Treatment strategies for mild cases often include oxygen therapy, rest, and close monitoring. Most cases resolving spontaneously within a week. However, significant cases necessitate intervention, such as needle decompression or closed drainage (7). Notably, while hyperbaric oxygen therapy is generally contraindicated in severe cases of pneumothorax and mediastinal emphysema (8), it may lead to gas compression of the heart and major blood vessels, and even endanger life. Some studies suggest that its potential efficacy in treating extra-pulmonary emphysema when other serious conditions are ruled out (9).

At high altitude, the challenges posed by limited medical facilities and the potential for misdiagnosis of extra-pulmonary emphysema as cardiovascular emergencies underscore the importance of accurate and timely diagnosis. This case report details the clinical presentation and management of nine patients diagnosed with extra-pulmonary emphysema at an altitude of 5,000 meters, highlighting the need for heightened awareness and appropriate treatment strategies in such environments. However, due to the small sample size and the different etiologies in each patient, the specific mechanisms still need to be further investigated.

## Case description

### Basic clinical information

This study included nine male patients diagnosed with subcutaneous emphysema or mediastinal emphysema who visited a high altitude medical station (at 3,700 meters) from May to December 2024. They all developed the condition in areas above 5,000 meters and were transported down to the medical station for further treatment. The basic information of the nine patients are shown in Tables 1, 2. None of them had a history of chronic diseases, nor did they have a history of surgery or immunosuppression. Before the onset of illness, three patients had never received oxygen therapy, two patients received oxygen therapy for 1 h daily, one patient for 2 h, one patient for 3 h, and two patients for 4 h. The duration of residence at altitude varied, ranging from 17 to 229 days. Upon arrival at the medical facility, patients reported discomfort including neck pain, chest pain, and chest tightness. Initial physical examinations revealed crepitus in some patients, with CT confirming the diagnosis of extra-pulmonary emphysema. Only two patients were diagnosed with isolated extra-pulmonary emphysema among all the patients, while the others had definite etiologies.

**Table 1:** Clinical summary of cases at high altitude.

No	Age (years)	Sex	HA (m)	Time at HA (days)	Oxygen inhalation time (h)	Primary diagnosis	Final diagnosis	Comorbidities	Treatment	Outcome
1	22	Male	5,100	30	0	SE	SE、ME	Aortic Dissection、DVT	Oxygen Inhalation (Mask) + Blood Pressure Control (Sodium Nitroprusside) + Heart Rate Control (Esmolol) + Symptomatic Supportive Treatment + Transfer to Plain Area + Surgical Treatment (Artificial Aortic Arch Replacement)	Improvement
2	23	Male	5,090	90	1	SE	SE	Cerebral Edema、Tracheal Rupture	Oxygen Inhalation (Non-invasive Ventilator) + Sedation (Propofol) + Dehydration (Furosemide + Human Albumin) + Steroids (Dexamethasone) + Symptomatic Supportive Treatment + Hyperbaric Oxygen Therapy (2.0 ATA)	Improvement
3	32	Male	5,100	94	4	SE	SE	Tracheal Rupture	Oxygen Inhalation (Mask) + Symptomatic Supportive Treatment + Hyperbaric Oxygen Therapy (2.0 ATA)	Improvement
4	21	Male	5,390	17	0	ME	ME	Pericarditis	Oxygen Inhalation (Mask) + Anti-infection (Imipenem/cilastatin) + Symptomatic Supportive Treatment + Hyperbaric Oxygen Therapy (2.0 ATA)	Improvement
5	20	Male	5,248	42	3	ME	ME	ME	Oxygen Inhalation (Mask) + Symptomatic Supportive Treatment + Hyperbaric Oxygen Therapy (2.0 ATA)	Improvement
6	22	Male	5,428	224	1	SE	SE	Acute Appendicitis	Oxygen Inhalation (Mask) + Anti-infection (Levofloxacin) + Surgical Treatment + Symptomatic Supportive Treatment + Hyperbaric Oxygen Therapy (2.0 ATA)	Improvement
7	33	Male	5,100	82	0	SE	SE	Pneumonia、Pulmonary Edema、Pulmonary Emphysema	Oxygen Inhalation (Mask) + Dehydration (Furosemide) + Steroids (Dexamethasone) + Anti-infection (Levofloxacin) + Symptomatic Supportive Treatment + Hyperbaric Oxygen Therapy (2.0 ATA)	Improvement
8	18	Male	5,100	229	4	ME	ME	ME	Oxygen Inhalation (Nasal Cannula) + Symptomatic Supportive Treatment + Hyperbaric Oxygen Therapy (2.0 ATA)	Improvement
9	21	Male	5,200	19	2	ME	ME	ICN	Oxygen Inhalation (Nasal Cannula) + Pain Relief (Ibuprofen) + Symptomatic Supportive Treatment + Hyperbaric Oxygen Therapy (2.0 ATA)	Improvement

SE: Subcutaneous Emphysema; ME: Mediastinal Emphysema; DVT: Deep Vein Thrombosis; BP: Blood Pressure; HR: Heart Rate; NIV: Non-Invasive Ventilation; HBOT: Hyperbaric Oxygen Therapy (2.0 ATA); ICN: Intercostal Neuralgia.

**Table 2:** Basic clinical characteristics of patients.

Variable	Mean or proportion
Age (years)	23.56 ± 5.27
Time at HA (days)	82 ± 24.5
Altitude (m)	5195.11 ± 133.36
Heart rate	100.44 ± 17.89
Systolic blood pressure	133.56 ± 29.09
Diastolic blood pressure	83.67 ± 11.42
Oxygen saturation	81.33 ± 6.56
Combined with pericarditis	11.1%
Combined with aortic dissection	11.1%
Combined with tracheal rupture	22.2%
Combined with upper respiratory infection	22.2%
Combined with pneumonia	11.1%
Combined with acute appendicitis	11.1%
Combined with cerebral edema	11.1%
Pulmonary edema	11.1%
Smoking	33.3%
Alcohol consumption	0
Immunodeficiency	0
Surgical history	0
Symptoms	
Cough	33.3%
Fever	44.4%
Neck and back pain	44.4%
Chest Pain	33.3%

Case 1 is 22 years old, he suddenly experienced severe neck pain while straining during a bowel movement. The pain persisted and radiated to the back. Six hours later, the patient was transferred to our department. His blood pressure was 202/103 mmHg in the right upper limb and 190/95 mmHg in another limb. The electrocardiogram showed “sinus rhythm, left axis deviation, left anterior fascicular block, and T-wave changes in the anterior leads.” Ultrasound revealed the formation of deep vein thrombosis. Non-contrast CT revealed emphysema in the muscles of the neck and back and subcutaneously, enhanced CT scan confirmed a thoracoabdominal aortic dissection. After stabilizing the blood pressure and heart rate, the patient was urgently transferred to a higher-level hospital for “aortic arch artificial vessel replacement.” Follow-up examinations indicated that the patient recovered well.

Case 2 and Case 3 are both young males. While engaging in heavy physical labor, both patients suddenly experienced chest pain. The patients were urgently transported to our medical facility and underwent chest CT, they were diagnosed with subcutaneous emphysema and tracheal rupture. Among them, case 2 also developed acute high altitude cerebral edema. After received symptomatic support and hyperbaric oxygen therapy (10, 11), the symptoms of these two patients significantly improved, and the extra-emphysema was also markedly absorbed. Follow-up showed that the tracheal rupture had also healed.

Case 4 is 21 years old. One week prior, he had a history of upper respiratory tract infection, and then He visited our hospital due to chest discomfort. The electrocardiogram suggested: “sinus rhythm, T-wave changes in the anterior leads,” and there was a slight increase in lymphocyte count. After underwent echocardiography and chest CT, he was diagnosed with mediastinal emphysema and pericarditis. In addition to antibiotic treatment, we also provided hyperbaric oxygen therapy (12, 13), and the patient eventually recovered well.

Case 6 is 22 years old, he experienced chest discomfort and abdominal pain after excessive alcohol consumption. After undergoing chest and abdominal CT, he was diagnosed with acute appendicitis and subcutaneous emphysema. Following an emergency “appendectomy,” he received hyperbaric oxygen therapy and had a good prognosis.

Case 7 is 33 years old, who developed symptoms of cough and chest pain after engaging in heavy physical labor outdoors. He was diagnosed with pneumonia and pulmonary edema through chest CT. Following this diagnosis, he received oxygen therapy, diuretics, antibiotic treatment, and hyperbaric oxygen therapy, his symptoms improved and he recovered well.

Case 5, Case 8, and Case 9 are all young males. All three patients presented with unexplained chest pain. Initially, primary healthcare institutions diagnosed them with cardiovascular emergencies and transferred them to our medical facility. After a series of examinations, including electrocardiogram, echocardiography, and chest and abdominal CT, Case 9 received a diagnosis of intercostal neuralgia, while Cases 5 and 8 were diagnosed with mediastinal emphysema. Follow-up after hyperbaric oxygen therapy showed significant resolution of mediastinal emphysema in all patients.

### Laboratory results and auxiliary

**Figure 1:**
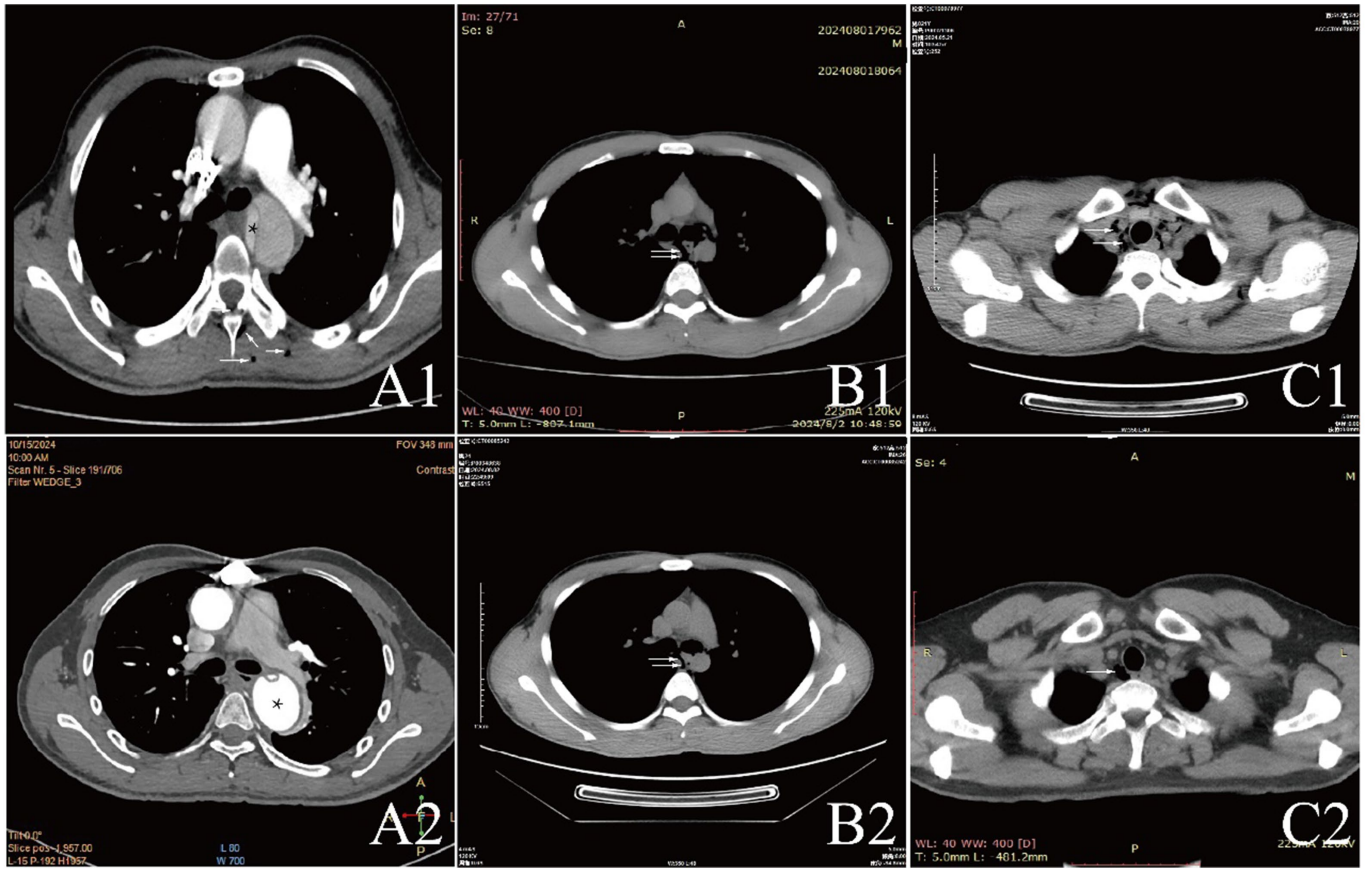
CT comparison images of extra-pulmonary emphysema treatment before and after for Case 1 to Case 3.

Examinations affected by the hypoxic environment of high altitudes, all patients exhibited varying degrees of increased red blood cell count, hemoglobin concentration, and uric acid (UA). Case 6 and 7, who were diagnosed with acute appendicitis and pulmonary infection, respectively, showed increased percentages of neutrophils. Case 1, in the acute phase of aortic dissection, also had an elevated white blood cell count. Additionally, we observed that D-dimer in the blood is a good indicator of myocardial or vascular damage. It showed varying degrees of elevation in patients with aortic dissection, while this indicator remained normal in other patients, maintaining a high negative predictive value. It is noteworthy that the serum potassium (K^+^) levels of all patients were high, with five patients having levels above the normal range. The average serum K^+^ level for all patients was 5.39 ± 0.73 mmol/L, this may be due to the increased metabolism in high altitude areas (Table 3). The diagnosis of extra-pulmonary emphysema for all patients was confirmed through CT examination. Case 1, 2, 3, 6, and 7 showed subcutaneous emphysema, while the others showed mediastinal emphysema. Specific imaging manifestations are shown in Figure 1.

**Table 3:** Laboratory test results of patients.

Variable	Case 1	Case 2	Case 3	Case 4	Case 5	Case 6	Case 7	Case 8	Case 9
RBC (10^12/L)	7.95	5.94	6.69	5.59	5.81	4.93	4.95	5.17	6.08
HB (G/L)	232	188	186	180	198	169	159	172	199
WBC (10^9/L)	12.9	6.26	8.5	8.13	8.04	14.06	6.98	9.8	7.6
Neut count (10^9/L)	11.74	3.82	4.44	4.87	5.43	12.05	5.72	6.78	5.22
Neut (%)	91	60.9	52.3	59.9	67.5	85.7	81.9	69.2	68.7
PLT (10^9/L)	119	198	350	243	160	287	159	335	177
CK (U/L)	58	125	199	80	261	95	94	90	75
CK-MB (U/L)	23.47	4.2	10.35	5.43	11.13	8.65	5.19	4.6	31.1
LDH (U/L)	347	244	339	221	233	272	126	312	159
BUN (mmol/L)	5.09	4.41	6.73	10.7	5.28	5.38	6.44	7.26	5.85
UA (umol/L)	443	345	478	473	454	357	457	170	483
Cr (umol/L)	63	100	63	106	85	83	95	81	94
D-Dimer (mg/L)	1.7	0.6	0.02	0.1	0.5	0.2	0.04	0.3	0.05
GPT (U/L)	30	17	41	20	25	42	21	24	13
GOT (U/L)	58	19	36	22	25	41	21	32	12
TBil (umol/L)	29.58	25.45	16.51	30.37	13.04	95.9	9.13	13.43	18.6
DBIL (umol/L)	4.08	15.77	2	5.89	3.51	13.19	2.21	3.12	4.49
IBIL (umol/L)	25.5	9.68	16.32	24.48	9.53	82.71	6.92	10.31	14.11
TP (g/L)	90.22	79.58	80.3	79.6	78.69	88.82	76.34	88.81	72.25
ALB (g/L)	54.62	54.38	49.69	51.9	44.3	52.31	48.71	48.42	45.94
GLB (g/L)	35.6	25.2	30.6	27.7	34.4	36.5	27.6	40.4	26.4
TG (mmol/L)	0.65	1.05	1.57	1.5	1.04	0.8	0.9	0.97	0.51
TC (mmol/L)	4.4	2.88	4.02	5.47	3	4.19	4.78	3.22	2.92
K^+^ (mmol/L)	5.84	4.55	5.3	5.52	5.68	5.76	4.68	6.7	4.46
Na^+^ (mmol/L)	136	139	138	136	137	131	152	124	144

RBC: Red Blood Cell Count; HB: Hemoglobin WBC: White Blood Cell Count; PLT: Platelet Count; BUN: Blood Urea Nitrogen; UA: Uric-Acid; Cr: Creatinine; GPT: Glutamic Pyruvic Transaminase; GOT: Glutamic Oxaloacetic Transaminase; TBil: Total Bilirubin; DBIL: Direct Bilirubin; IBIL: Indirect Bilirubin; TP: Total Protein; ALB: Albumin; GLB: Globulin; TG: Triglyceride; TC: Total Cholesterol.

**Figure 2:**
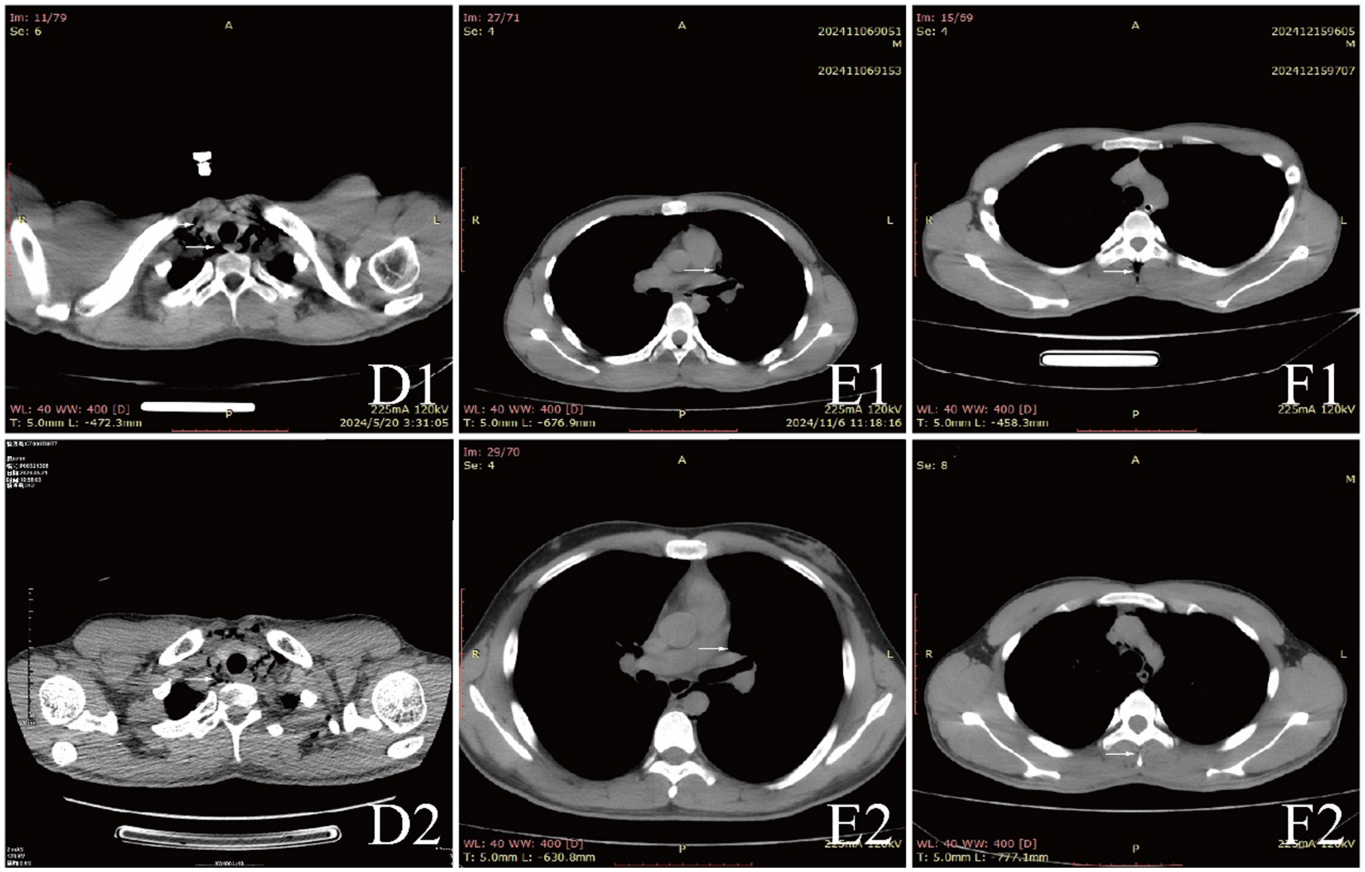
CT comparison images of extra-pulmonary emphysema treatment before and after for Case 4 to Case 6.

### Diagnosis, treatment and prognosis

**Figure 3:**
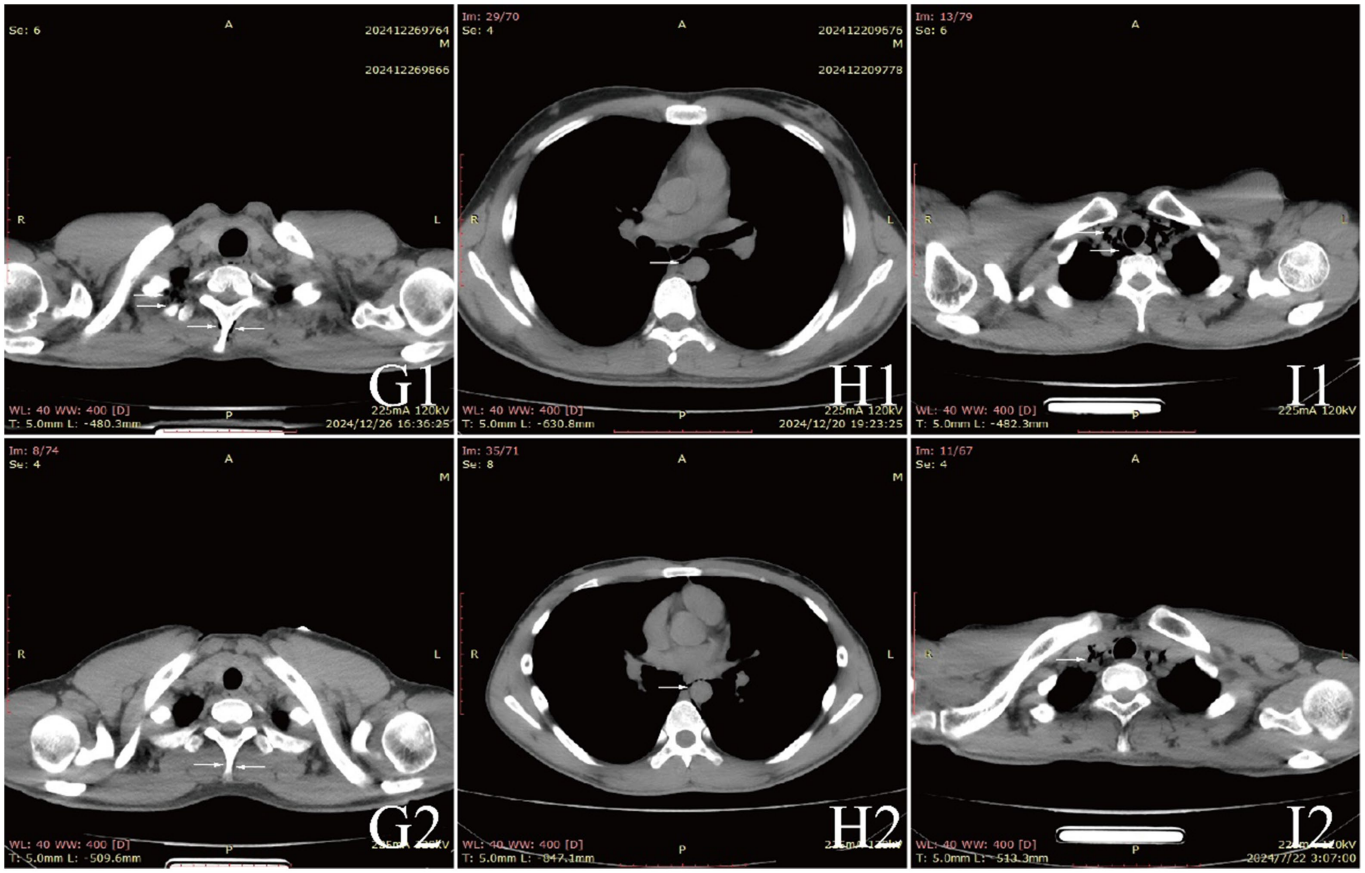
CT comparison images of extra-pulmonary emphysema treatment before and after for Case 7 to Case 9.

All patients were diagnosed with extra-pulmonary emphysema through CT scans, which showed gas infiltration in the mediastinum or subcutaneous tissue. The patient with aortic dissection was urgently transferred to a lower altitude area for “aortic arch artificial vessel replacement surgery.” Case 6 underwent “appendectomy” for treatment, and three patients received antibiotic therapy. All patients were given symptomatic and supportive treatment, including oxygen therapy. Six were given oxygen via a face mask, two received a nasal cannula, and one was assisted with non-invasive ventilation. In addition to case 1, they also received hyperbaric oxygen therapy at 2.0 ATA. Follow-up showed that all patients responded well to the treatment, their conditions improved, and the emphysema was largely absorbed (Figures 1–3).

## Discussion

The occurrence of extra-pulmonary emphysema, particularly at high altitudes, represents a significant clinical challenge due to its rarity and potential for misdiagnosis (14–17). In our retrospective analysis of patients treated for this condition at altitudes exceeding 5,000 meters, we observed a concerning trend: many patients initially presented with symptoms that could easily be mistaken for cardiovascular emergencies, such as aortic dissection and pulmonary embolism. This underscores the critical need for heightened awareness and understanding of extra-pulmonary emphysema among healthcare providers, particularly in high-altitude settings where diagnostic resources may be limited. In the special environment of the plateau, medical resources are scarce, and most areas may only be equipped with X-ray examinations without conducting CT scans. This may lead to two types of misdiagnoses: either mistaking simple emphysema for cardiovascular emergencies such as pulmonary embolism or aortic dissection, or diagnosing emphysema solely based on X-ray examination while overlooking atypical critical illnesses. Both of these can result in excessive or insufficient treatment strategies. CT scans, through multiplanar imaging and 3D reconstruction analysis, can effectively avoid these situations. Through CT sections, we can observe vascular filling defects or intimal tears, thereby effectively obtaining diagnostic information for conditions such as pulmonary embolism and aortic dissection, which can guide subsequent medical procedures.

In our series, the majority of patients were young males with no significant medical histories, indicating that even previously healthy individuals can be susceptible to extra-pulmonary emphysema under extreme environmental stressors (18, 19). The clinical presentation varied, with symptoms such as neck pain, chest tightness, and crepitus upon examination. However, the diagnosis was often missed or delayed in primary care settings, reflecting a gap in knowledge regarding the manifestations of this condition at high altitudes. The initial misdiagnoses highlight a critical area for improvement in clinical practice, emphasizing the importance of considering extra-pulmonary emphysema in differential diagnoses for patients presenting with chest pain or dyspnea in such environments.

Furthermore, our findings support the efficacy of hyperbaric oxygen therapy as a treatment modality for extra-pulmonary emphysema. Eight out of nine patients showed improvement following this intervention, illustrating its potential in expediting recovery in high-altitude scenarios (20–22). Most cases of extra-pulmonary emphysema are asymptomatic or mildly symptomatic, and the standard treatment is typically the administration of pure oxygen under normal pressure. However, if hyperbaric oxygen therapy is administered, the volume of gas in the alveoli will change with the increase and decrease of ambient pressure during treatment. If the gas in bullae or air cysts cannot be normally expelled, it may lead to alveolar overexpansion and even rupture, thereby causing rare but serious complications such as pneumothorax and gas embolism. At the same time, the accumulation of a large amount of gas can affect the return flow of venous blood, leading to thrombotic diseases such as deep vein thrombosis or pulmonary embolism. In addition, if a patient with emphysema also has an untreated pneumothorax, this is an absolute contraindication for hyperbaric oxygen therapy, as the pneumothorax may rapidly worsen during treatment, leading to severe respiratory and circulatory dysfunction. Therefore, before undergoing hyperbaric oxygen therapy, patients with extra-pulmonary emphysema must undergo a comprehensive evaluation by a professional physician. During the treatment process, the general condition of the patient needs to be closely monitored to reduce the risk of pulmonary complications.

This analysis also draws attention to the limitations of medical facilities in high-altitude regions, where access to advanced imaging and definitive diagnostic capabilities may be restricted. The challenges of accurate diagnosis and timely intervention are compounded by the geographical isolation of many high-altitude communities, making early recognition of conditions such as extra-pulmonary emphysema crucial. Future studies should focus on developing standardized protocols for the diagnosis and management of extra-pulmonary emphysema in these settings, as well as exploring the long-term outcomes of patients treated at high altitudes.

In conclusion, our findings highlight the need for increased clinical awareness of extra-pulmonary emphysema, especially in high-altitude environments, where its presentation can mimic more serious cardiovascular conditions. Enhanced understanding and recognition of this condition may improve patient outcomes, as timely diagnosis and appropriate management strategies, such as hyperbaric oxygen therapy, can be life-saving (23–25). The lessons learned from our cases should inform future clinical practice and guide the development of emergency protocols for healthcare providers operating in high-altitude regions.

The limitations of this study are that the number of cases was small, and we do not have a control group. Meanwhile, after reviewing the relevant literature, we found that plasma sRAGE, CT images analyzed by deep learning, and blood-based transcriptomic and proteomic biomarkers can all serve as early warning indicators for the development of emphysema (26–28). Unfortunately, due to the limitations of our medical environment, we were unable to collect biological samples from the patients and thus could not conduct the relevant tests. In future work, we can pay more attention to research in this area.

## Conclusion

In this study, a retrospective analysis of extra-pulmonary emphysema case data in extreme high altitude areas suggested that the diagnosis of extra-pulmonary emphysema should be emphasized in high altitude regions. After ruling out fatal diseases, administering hyperbaric oxygen therapy and symptomatic treatment is a reliable approach.

## Data Availability

The original contributions presented in the study are included in the article/supplementary material, further inquiries can be directed to the corresponding author/s.
